# Severe Group A *Streptococcus* Infection among Children, France, 2022–2024

**DOI:** 10.3201/eid3109.250245

**Published:** 2025-09

**Authors:** Montserrat Sierra Colomina, Alix Flamant, Guillaume Le Balle, Jérémie F. Cohen, Lionel Berthomieu, Stéphane Leteurtre, Yves Gillet, Etienne Javouhey, Stéphane Bechet, Corinne Levy, Robert Cohen, Agnès Ferroni, Damien Dubois, Miguel Angel Hernandez Martinez, Céline Plainvert, Asmaa Tazi, ISAI Study Group,, Camille Brehin

**Affiliations:** CHU Toulouse, Toulouse, France (M. Sierra Colomina, G. Le Balle, L. Berthomieu, D. Dubois, C. Brehin); Necker-Enfants Malades Hospital, Assistance Publique-Hôpitaux de Paris, Paris, France (A. Flamant, J.F. Cohen, A. Ferroni); Lille University, CHU Lille, Lille, France (S. Leteurtre); Groupe Francophone de Réanimation et Urgences Pédiatriques, Lille (S. Leteurtre); CHU Lyon, Lyon, France (Y. Gillet, E. Javouhey); Association Clinique et Thérapeutique du Val de Marne, Créteil, France (S. Bechet, C. Levy); Groupe de Pathologie Infectieuse Pédiatrique, Créteil (R. Cohen); National Reference Centre for Streptococci, Assistance Publique-Hôpitaux de Paris, Paris Centre, Hôpital Cochin, Paris (M.A. Hernandez Martinez, C. Plainvert, A. Tazi)

**Keywords:** group A streptococcus, GAS, Streptococcus pyogenes, bacteria, streptococci, respiratory infections, invasive infection, pediatrics, France

## Abstract

Group A *Streptococcus* infections have increased in Europe since September 2022. The French Pediatric Intensive Care and French Pediatric Infectious Diseases expert groups conducted a retrospective and prospective study of children who had severe group A *Streptococcus* infections during September 1, 2022–April 1, 2024, across 34 hospitals in France. A total of 402 pediatric patients (median age 4 [interquartile range 2–7.5] years; 42% girls, 58% boys) were enrolled. Cases were characterized by a low proportion of severe skin and soft tissue infections (16%), predominance of severe upper and lower respiratory tract infections (55%), and a 3.5% case-fatality rate. In multivariate analysis, hydrocortisone, corticosteroid, and vasopressor therapies were significantly associated with major sequelae or death. Molecular analysis revealed *emm1* (73.0%) and *emm12* (10.8%) strains; the M1_UK_ clone represented 50% of *emm1* strains. Clinicians, researchers, and public health authorities must collaborate to mitigate the effects of GAS on child health.

*Streptococcus pyogenes*, also known as group A *Streptococcus* (GAS), presents a wide spectrum of manifestations, ranging from mild infections (e.g., pharyngitis) to severe and life-threatening conditions (e.g., necrotizing fasciitis). Globally, invasive *S. pyogenes* infections (iGAS) account for nearly 2 million cases per year worldwide; the effects of those infections on young children and older adults are substantial, including a case-fatality rate of up to 20%. In late 2022 and early 2023, a surge in pediatric GAS infections garnered international attention, prompting public health agencies to issue alerts. This rise extended beyond benign cases, also encompassing a notable increase in iGAS cases (*1*–*14*).

In France, during the last 2 weeks of November 2022, pediatric clinicians and intensivists reported a higher than usual number of pediatric cases of iGAS to the French Public Health (Santé publique France) and the Regional Healthcare Agencies (Agences Régionales de Santé). The National Reference Center for Streptococci also observed an increase in the number of GAS strains received since the summer of 2022 compared with previous years, particularly involving strains isolated from severe pediatric cases as of the end of October 2022. Those GAS infections occurred in different regions in France and mainly in children <10 years of age. In response, Santé publique France and its partners (the Groupe de Pathologie Infectieuse Pédiatrique, Association Clinique et Thérapeutique du Val de Marne, and the Groupe Francophone de Réanimation et Urgences Pédiatriques) have set up surveillance of these infections.

The question of the emergence of a hypervirulent GAS clone immediately arose, as did the search for risk factors associated with the most severe forms. The primary objective of this study was to describe the clinical, biological, and microbiological characteristics of pediatric severe GAS cases that occurred in France during the 2022–2023 epidemic. Our secondary objectives were to identify risk factors associated with the most severe clinical forms, describe complications and short-term outcomes, and describe the treatment and short-term management of severe pediatric GAS cases.

## Materials and Methods

### Study Population and Data Collection

We conducted a retrospective (September 1, 2022–December 31, 2022) and prospective (January 1, 2023–April 30, 2024) national, multicenter cohort study in 34 hospitals in France. We included all hospitalized pediatric case-patients <18 years of age. 

We included all cases of severe GAS infections, which included proven and probable iGAS cases. Proven iGAS was defined as per international criteria: cases in which GAS was isolated from a normally sterile site (by culture, PCR, or rapid antigen detection testing), such as blood, cerebrospinal fluid, pleural fluid, peritoneal fluid, pericardial fluid, bone, joint/synovial fluid, or internal body site (e.g., lymph node, brain); or GAS isolated from a nonsterile site (such as a wound) and accompanied by necrotizing fasciitis or streptococcal toxic shock syndrome (*15*). We also included probable iGAS, defined as GAS isolated from a nonsterile site such as sputum or otorhinolaryngology surgical specimens (mastoiditis, ethmoiditis, pharyngeal abscess) accompanied by >1 of the following severity criteria: intravenous (IV) antibiotic drugs, surgery, or admission to the pediatric intensive care unit (PICU). Clinical data collected were demographic information (age, sex, town of residence, number of siblings, comorbidities, vaccination status, and allergies), mode of onset of illness (fever, history of viral infection within the previous 15 days), clinical manifestations, clinical evaluation upon admission, biologic and microbiological data, drug therapy, in-hospital evolution, and patient outcomes (Appendix).

### Microbiological Workup

We performed local microbiological documentation at each participating center using culture, GAS-specific PCR, 16S rDNA PCR, and antigen detection testing, as part of usual care. After inclusion of a case in the study, we sent GAS isolates to the National Reference Center for Streptococci for further characterization. Isolates were confirmed as GAS on the basis of colony morphology on horse blood agar and by matrix-assisted laser desorption/ionization time-of-flight mass spectrometry (Bruker Daltonics). We determined the *emm* genotype of each strain by sequencing the variable 5′ end of the *emm* gene and comparing sequences with the database of the US Centers for Disease Control and Prevention (https://www2.cdc.gov/vaccines/biotech/strepblast.asp). We also identified the toxin profile, particularly for superantigenic toxins. We performed antibiotic susceptibility testing by disc diffusion according to the European Committee on Antimicrobial Susceptibility Testing (http://www.eucast.org). We performed a double-disc diffusion test to detect inducible resistance to clindamycin. We used PCR to detected genetic determinants of resistance to aminoglycosides; macrolides, lincosamides, and synergistins; and tetracycline, as previously described (*16*).

We performed whole-genome sequencing on a sample of randomly selected GAS strains. We extracted genomic DNA from overnight cultures using the MasterPure Gram Positive DNA Purification kit (LGC Biosearch Technologies) according to the manufacturer’s recommendations. We sequenced the DNA using the Illumina MiSeq system. Bioinformatical analyses included identification of the M1_UK_ clone, by recognizing its specific 27 single-nucleotide polymorphisms (*17*).

### Statistical Analysis

First, we described included cases using standard statistics. We expressed qualitative variables as frequencies and percentages and quantitative variables using the median and interquartile range (if not normally distributed). For the comparisons between groups (i.e., group without sequelae or minor sequelae vs. group with major sequelae or death; group with hospitalization in Pediatric Intermediate Care Unit [PIMCU]/PICU vs. group without hospitalization in PIMCU/PICU), we compared percentages using the χ^2^ or Fisher exact test, depending on the number of patients. We compared quantitative variables using a Mann-Whitney test. We conducted all tests with a significance threshold of p<0.05.

Second, to identify risk factors for the most severe cases, we performed multivariate analysis using logistic regression models. A first model aimed to identify risk factors for admission in PIMCU/PICU, and a second model focused on identifying risk factors for major sequelae or death. We included all variables with a p value <0.20 on univariate analysis in the multivariable models and removed them one by one using a stepwise backward elimination approach, with a threshold of p<0.05, to obtain the final models. We conducted the analyses using Stata 18 (StataCorp).

### Ethics Statement

In accordance with France’s ethics law, patients were informed that their encoded data would be used for the study and for publication. Their nonopposition to the use of their data and publication was collected. Patients were not involved in the design of this study. This study is registered in ClinicalTrials.gov as NCT05788861 and has been approved by the ethics review board of the Créteil Hospital Center (Créteil, France).

## Results

### Study Population

**Figure 1:**
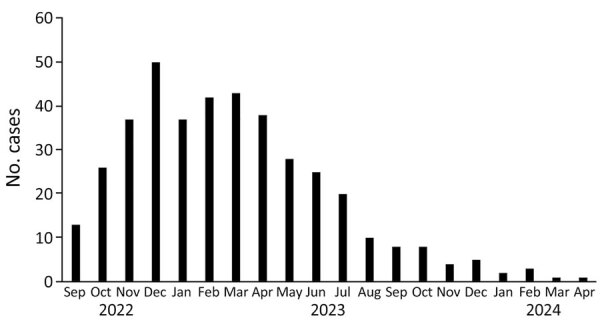
Trends in the number of hospital admissions for severe group A *Streptococcus* infection among 402 children, by month, France, 2022–2024.

**Figure 2:**
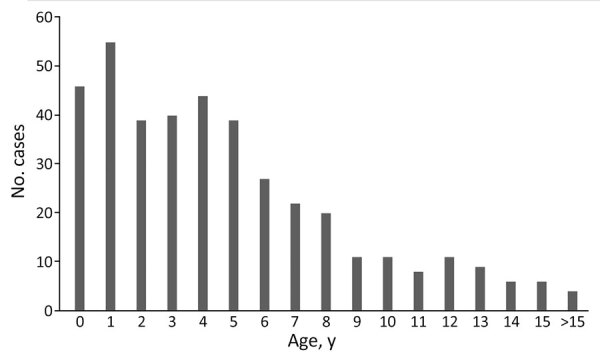
Severe group A *Streptococcus* infection distribution in 402 children by age, France, 2022–2024.

We enrolled a total of 402 pediatric patients with severe GAS infections. Median age was 4 (interquartile range [IQR] 2–7.5) years; 42% were girls and 58% boys (Table 1; Figures 1 and 2). The most frequent comorbidities were chronic pulmonary diseases (2.2%; 9/402), notably asthma (7/9 cases). Approximately 2% of the cohort (7/402) were immunocompromised children (3 with primary immunodeficiency and 4 receiving immunosuppressive drugs). Regarding drug exposure within 7 days before admission, 8% (34/402) had recently received nonsteroidal antiinflammatory drugs (NSAIDs), 4% (17/402) had received steroids, and 25% (100/402) had received antibiotic drugs. Most GAS infections were isolated cases (81%, 326/402); 5 (1%) cases were identified as associated with collective settings, and 27 (7%) cases were linked to household clusters. A substantial percentage of children (44%) were admitted to general pediatric wards, 23% required admission to the PICU, 7% were managed in PIMCUs, and 29% were hospitalized in other specialized wards, such as neurology and surgery departments.

**Table 1:** Clinical features of severe group A *Streptococcus* infections among 402 children, France, 2022–2024*

Clinical features	Value
Median age (IQR), y	4 (2–7.5)
Sex	
M	234 (58)
F	168 (42)
Comorbidities	52 (13)
Immunosuppression	7 (2)
Viral infection in the previous 15 d	109 (27)
Influenza	28/112 (25)
Respiratory syncytial virus	7/112 (6)
Chicken pox	37/112 (33)
SARS-CoV-2	5/112 (4)
Other	37/112 (33)
Isolated case	326 (81)
Isolated fever	18 (4)
Site of infection	
Multiple	146 (36)
Ear-nose-throat	135 (34)
Skin and soft tissue	63 (16)
Bone and joint	57 (14)
Lower respiratory	84 (21)
Pleuro-pneumonia	69 (17)
Others†	34 (8)
Septic shock‡	34 (8)
Toxic rash	37 (9)

*Values are no. (%) except as indicated. †Meningitis, endocarditis, peritonitis, dental cellulitis. ‡Defined as persistence of several organ failures or acute circulatory failure, failure to correct arterial hypotension despite vascular filling of >40 mL/kg, or both.

**Figure 3:**
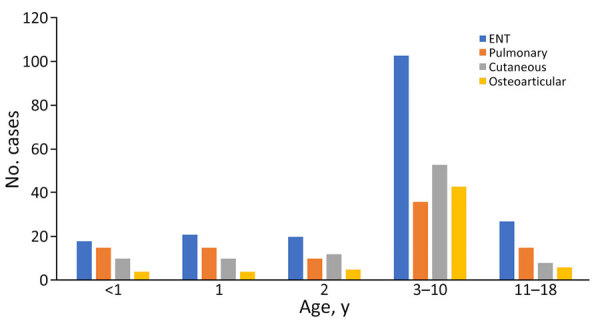
Distribution of severe group A *Streptococcus* infection in 402 children, by type of infection and age, France, 2022–2024. ENT, ear-nose-throat.

A total of 140 patients were <3 years of age, and the most frequent clinical manifestation was ear-nose-throat (ENT) damage (Table 2; Figure 3). One patient experienced out-of-hospital cardiac arrest, and 3% (12/402) of patients had acute respiratory distress syndrome. Acute respiratory distress was prevalent in 27% (108/402) of cases; 78% (84/108) of those were categorized as mild and 22% (24/108) as severe. During hospital stay, 3% (12/402) of patients experienced a cardio-respiratory arrest. Median C-reactive protein level at admission was 161 (IQR 76–254) mg/L (reference range 0–5 mg/L).

**Table 2:** Management of severe group A *Streptococcus* infections among 402 children, France, 2022–2024

Treatment	No. (%)
Oxygen therapy	120 (30)
Conventional	45 (11)
High-flow	27 (7)
Noninvasive ventilation	11 (3)
Invasive ventilation	32 (8)
Extracorporeal membrane oxygenation	5 (1)
Hemodynamic therapy	97 (24)
Fluid resuscitation	97 (24)
Vasopressor treatment	50 (12)
First-line antibiotic treatment	
Amoxicillin/clavulanate	138 (34)
Third generation cephalosporin	141 (35)
β-lactam + clindamycin	173 (43)
Adjunctive therapies	
Intravenous Immunoglobulins	17 (4)
Hydrocortisone hemisuccinate for shock	19 (4)

Nearly half of patients (44% [177/402]) required surgical intervention during their hospital stay; causes consisted of ENT drainage in 41% (73/177), osteoarticular drainage in 24% (43/402), and deep tissue abscess drainage in 6% (23/177) of cases (Table 2). Surgical intervention within the first 24 hours was necessary in 29% (117/402) of cases, primarily for procedures such as deep tissue pus drainage and joint drainage. Median duration of hospital stay was 7 (IQR 3–12) days.

### Bacterial Data

Rapid antigen detection tests showed high positivity rates when performed; results were positive for 79% (110/139) of throat samples, 70% (48/69) of pleural effusion samples, and 39% (13/33) of cutaneous samples. PCR testing identified GAS DNA in 14% of cases, predominantly in pleural effusion fluid (38%, 22/58), followed by deep tissue abscesses (especially from ear, nose, and throat area) (32%, 19/58). GAS culture yielded positive results in 71% (285/402) of cases; GAS was isolated from blood cultures in 21% (84/402) of cases.

A total of 149 GAS isolates were sent to the National Reference Center for Streptococci (Table 3), of which 148 underwent *emm* typing. The *emm1* (73.0%, 108/148) and *emm12* (10.8%; 16/148) genotypes represented most strains. Resistance to macrolides (defined by erythromycin resistance) was found in 3% (5/148) of GAS strains. All macrolide-resistant isolates were also resistant to clindamycin.

**Table 3:** Molecular characterization of 149 *Streptococcus pyogenes* isolates analyzed at the French National Reference Center for Streptococci in study of children with severe group A *Streptococcus* infections, France, 2022–2024

Variable	No. (%)
Superantigen genes	148
SpeA	110 (74.3)
SpeB	148 (100)
SpeC	76 (51.3)
SmeZ	146 (98.6)
Sic*	108 (73.0)
ssa	11 (7.4)
*emm* genotypes	148
* emm1*	108 (73.0)
* emm4*	4 (2.7)
* emm12*	16 (10.8)
* emm77*	4 (2.7)
* emm87*	4 (2.7)
* emm89*	4 (2.7)
Others†	8 (5.4)
Not done	1
Whole-genome sequencing	25
* emm1*	20
M1_UK_ clone among *emm1* strains	10

*Sic-like genes were not analyzed. †Others: 1 strain of each of *emm2*, *emm3*, *emm11*, *emm22*, *emm44*, *emm75*, *emm169*, and *emm209*.

We performed whole-genome sequencing on 25 of the 149 isolates received at the National Reference Center for Streptococci, including 20 *emm1* isolates. The M1_UK_ clone represented 50.0% (10/20) of the *emm1* isolates.

### Outcomes and Sequelae

Most (77%, 311/402) patients affected by severe GAS infections were discharged with a favorable outcome, devoid of sequelae. However, 17 (4%) patients had major sequelae (amputation/cutaneous necrosis/orthopedic sequelae [n = 7], impaired respiratory function [n = 6], neurologic deficit [n = 4]), and 14 deaths were recorded, resulting in a case-fatality rate of 3.5%.

Initial clinical factors significantly associated with hospitalization in the PICU or PIMCU in univariate analysis included age <3 years, exposure to corticosteroids in the days before hospitalization, viral infection within 15 days before symptom onset, type of disease, and presence of a toxic rash (Table 4). In multivariate analysis (n = 399), factors significantly associated with hospitalization in the PICU or PIMCU consisted of viral infection within the 15 days before symptom onset (adjusted odds ratio [aOR] 2.0 [95% CI 1.2–3.4]; p = 0.007) and type of disease (bone and joint damage, aOR 0.3 [95% CI 0.1–0.8], p = 0.013; pulmonary damage, aOR 2.1 [95% CI 1.04–4.3], p = 0.037) (Table 4).

**Table 4:** Univariate and multivariate analysis of factors associated with PIMCU or PICU hospitalization in children with severe group A *Streptococcus* infections, France, 2022–2024*

Category	PIMCU/PICU hospitalization	No PIMCU/PICU hospitalization	p value
Univariate analysis			
Age group, y			
<1	18/117 (15.4)	33/284 (11.6)	**0.031**
1	11/117 (9.4)	39/284 (13.7)	
2	19/117 (16.2)	20/284 (7)	
3–10	55/117 (47)	159/284 (56)	
11–18	14/117 (12)	33/284 (11.6)	
Immunosuppression	3/115 (2.6)	4/282 (1.4)	0.414
Comorbidities	16/116 (13.8)	36/283 (12.7)	0.773
Exposure to nonsteroidal antiinflammatory drug	13/112 (11.6)	21/272 (7.7)	0.223
Exposure to steroidal antiinflammatory drug	14/112 (12.5)	3/275 (1)	**<0.001**
Viral infection in the 15 days before hospitalization	47/109 (28.9)	62/268 (23.1)	**<0.001**
Type of infection			
ENT	31/117 (26.5)	104/284 (36.6)	**<0.001**
Pulmonary	44/117 (37.6)	40/284 (14.1)	
Bone and joint	7/117 (6)	50/284 (17.6)	
Cutaneous	16/117 (13.7)	47/284 (16.5)	
Other	19/117 (16.2)	43/284 (15.1)	
Multiple damage	50/117 (42.7)	96/284 (33.8)	0.091
Toxic rash	17/117 (14.5)	20/284 (7)	**0.019**
Multivariate analysis, adjusted odds ratio (95% CI)			
Viral infection in the 15 days before hospitalization	2.02 (1.21–3.37)		0.07
Osteoarticular damage	0.29 (0.11–0.77)		**0.013**
Pulmonary damage	2.11 (1.04–4.29)		**0.037**

*Values are no. (%) except as indicated. Boldface indicates significance. ENT, ear-nose-throat; PICU, pediatric intensive care unit; PIMCU, pediatric intermediate care unit.

Factors significantly associated with major sequelae or death in univariate analysis were a viral infection within 15 days before symptom onset, hospitalization in PIMCU or PICU, antibiotic therapy with a β-lactam combined with clindamycin, receipt of IV immunoglobulins, receipt of hydrocortisone, and receipt of vasopressor therapy (Table 5). In multivariate analysis (n = 395), only hydrocortisone, use of corticosteroids during hospitalization, and vasopressor therapy remained significantly associated with major sequelae or death (Table 4).

**Table 5:** Univariate and multivariate analysis of factors associated with major sequelae/death in children with severe group A streptococcus infections, France, 2022–2024*

Category	Major sequelae/death	No major sequelae/death	p value
Univariate analysis			
Female sex	14/31 (45)	150/365 (41)	0.668
Viral infection in the 15 days before hospitalization	16/31 (52)	88/365 (24.1)	**0.004**
Clinical damage			0.094
ENT	8/31 (25.8)	127/365 (34.8)	
Pulmonary	11/48 (35.5)	69/365 (18.9)	
Osteoarticular	3/31 (9.7)	54/365 (14.8)	
Cutaneous	2/31 (6.5)	60/365 (16.4)	
Other	7/31 (22.6)	55/365 (15)	
PIMCU/PICU admission	22/31 (71)	90/365 (24.7)	**<0.001**
Clindamycin therapy	21/31 (67.7)	148/365 (40.5)	**0.003**
Intravenous immunoglobulins	6/31 (19.3)	10/355 (2.8)	**<0.001**
Corticosteroidal therapy	5/28 (17.9)	36/356 (10.1)	0.201
Hydrocortisone hemisuccinate for shock	12/29 (41.4)	7/353 (1.9)	**<0.001**
Vasopressive drug	19/30 (63.3)	25/343 (7.3)	**<0.001**
Multivariate analysis, odds ratio (95% CI)			
Corticosteroidal therapy	4.18 (1.27–13.75)		**0.018**
Hydrocortisone hemisuccinate for shock	6.25 (1.53–25.50)		**0.011**
Vasopressive drug	11.27 (3.54–35.89)		**<0.001**

*Values are no. (%) except as indicated. Boldface indicates significance. ENT, ear-nose-throat; PICU, pediatric intensive care unit; PIMCU, pediatric intermediate care unit.

Genotypes *emm1* and *emm12* were not significantly associated with the risk for admission to PIMCU/PICU or the risk for persistent sequelae. Genotype *emm1* was significantly associated with respiratory damage (upper and lower) compared with other types of damage (p = 0.01).

## Discussion

This study provides detailed characterization of severe GAS infections in pediatric patients in France. It covers a wide range of clinical data, including demographic characteristics, clinical manifestations, laboratory findings, treatment modalities, and outcomes, thereby providing a thorough understanding of the disease spectrum and its implications. Key findings include the absence of emerging GAS clones, a predominance of *emm1* and *emm12* genotypes, a case-fatality rate of 3.5%, and a predominance of upper and lower respiratory tract infections. We were also able to identify several risk factors associated with the most severe forms of GAS disease.

One third of severe infections occurred in children <3 years of age, an age group in which benign GAS infections (mainly ENT-related) are considered rare, underlining the challenges of early diagnosis in this age group. The frequency of acute GAS infections peaks in childhood, but invasive infections are observed in the extreme age groups (<1 and >65 years of age) (*18*). This pattern could be explained by immune system naivety in young children and immunosenescence in the elderly. The strongest evidence for the existence of protective immune responses against GAS is the observed decrease in susceptibility to infection with increasing age. The incidence of symptomatic throat infections, including scarlet fever, increases dramatically around the age of 4 (*19*). This increase could be caused by the expansion of tonsil tissue, which enables better access to GAS; entry into school communities, with increased exposure to GAS; or simply by the ability of children of that age to verbalize sore throats. Toward the end of childhood, the frequency of streptococcal pharyngitis decreases substantially. GAS skin infections exhibit a similar peak and fall in incidence but at a slightly earlier age than throat infections, perhaps linked to a greater frequency of skin lesions in young children (*20*).

In our cohort, clinical manifestations consisted predominately of ENT and lung infections, consistent with findings from other studies (*7*). The 2022–23 winter in France saw exceptionally intense viral epidemics, including respiratory syncytial virus, influenza, and SARS-CoV-2, compared with previous years. Such respiratory viral infections have been associated with increased susceptibility to severe GAS infections and related ENT and pulmonary complications, suggesting a potential interplay between viruses and bacteria (*21*,*22*). Varicella zoster virus has also been implicated in both cutaneous and articular manifestations of iGAS infections, further supporting a potential synergistic relationship that might exacerbate disease severity (*23*,*24*). Those findings highlight the importance of monitoring viral coinfections and their potential role in the pathogenesis of iGAS.

The concept of an immunity debt because of reduced exposure and heightened hygiene measures during COVID-19 lockdowns has been proposed to explain the resurgence in 2022–2023 of invasive bacterial infections in children, including pneumococcal, meningococcal, and pertussis infections (*2*,*25*,*26*). Similarly, a rise in mild GAS infections in outpatient settings was reported in France before the 2022–2023 surge in severe cases (*2*). However, our study cannot confirm this hypothesis for iGAS infections, given the absence of pre-2022 data (Appendix) and the uncertainty regarding a potential continuum between noninvasive and severe forms of the disease, despite the high incidence of severe ENT infections and pneumonia observed in our cohort (*23*). Those considerations underscore the need for robust outpatient surveillance and appropriate use of diagnostic tools to guide GAS management. In addition, considering COVID-19 infection itself as a potential predisposing factor for iGAS is critical, as previously discussed.

The case-fatality rate of 3.5%, the elevated surgical intervention rate (44%), and the long median of hospital stay (7 days) illustrate the burden of severe GAS infections on patients, their families, and the healthcare system. Although GAS is often considered a minor pathogen responsible for mostly mild infections, the 2022–2023 worldwide epidemic reminds us that GAS possesses a full range of virulence factors that are capable of causing severe damage and must not be overlooked. In low- and-middle income settings, rheumatic fever is an additional concern. Those challenges have prompted clinicians to diagnose and treat noninvasive infections, such as pharyngitis, and has prompted researchers to develop vaccines against them. Of note, the promising 30-valent M protein–based vaccine described by Finn et al. (*27*) includes almost all the *emm* types identified in our study. It covers the predominant *emm* types circulating in temperate regions, highlighting its potential for broad epidemiologic coverage.

Further research is needed to establish the most effective therapeutic strategies for managing iGAS infections. The role of adjunctive treatments such as clindamycin, which might help reduce GAS toxin production, and IV immunoglobulin, which might neutralize toxin activity, remains a subject of debate. A recent observational study from Japan found no significant effect of those therapies on in-hospital mortality (*22*). In our own multivariate analysis, only the use of hydrocortisone, corticosteroids, and vasopressor therapy during hospitalization was significantly associated with major sequelae or death. However, those findings likely reflect their use in the most severely ill patients, rather than indicating a direct causal effect.

The most frequent genotypes identified in our pediatric cohort were *emm1* and *emm12*, similar to those reported in other countries during the 2022–2023 epidemic and in earlier prepandemic cohorts (*24*,*28*). However, the proportion of *emm1* strains in our series was higher than in other studies (e.g., Spain, United Kingdom, Portugal series) (*5*,*29*–*31*). Of note, a recent population-based study conducted in 10 US states that included 21,312 patients (1,272 children) with invasive GAS infections during 2013–2022 documented a decline in pharyngeal strains (such as *emm1* and *emm12*) during the COVID-19 pandemic, alongside an increase in skin-associated *emm* types (*24*). The epidemic in France confirmed the continued expansion of the M1_UK_ clone, which has been progressively spreading since its first detection in the United Kingdom in 2008 (*17*).

The first limitation of our study is that, despite our thorough description of the severe GAS infections observed during the 2022–2023 outbreak, the lack of a control period from the pre–COVID-19 era prevents us from drawing precise epidemiologic conclusions about changes in incidence rates over time (Appendix). Second, the relatively small number of isolates and the limited subset analyzed by whole-genome sequencing further restrict our ability to detect the emergence of specific strains. Finally, although this study relies on a large and nationwide network of 34 hospitals, we cannot guarantee the exhaustiveness or full representativeness of our case series.

Future research endeavors should focus on elucidating the underlying mechanisms driving the surge in pediatric severe GAS infections and the interplay between viral co-infections and bacterial complications. Comprehensive surveillance programs targeting both viral and bacterial pathogens, including GAS, are essential for early detection, timely intervention, and prevention of severe GAS disease outcomes. Furthermore, enhanced understanding of host–pathogen interactions, immune responses, genetic predispositions, and pathogen virulence that influence susceptibility to severe GAS infections is needed. Such knowledge can inform the development of targeted therapeutic approaches, including immunomodulatory strategies and vaccine development, to mitigate the burden of iGAS infections in pediatric populations.

In conclusion, pediatric severe GAS infections represent a substantial clinical and public health challenge, characterized by a high number of cases and associated illnesses and deaths during the winter of 2022–23 in several countries. This cohort in France demonstrated a high rate of respiratory diagnoses and a significant need for procedural intervention, mainly driven by *emm1* and *emm12* genotypes and the M1_UK_ strain. Advances in diagnostic modalities, antibiotic therapy, and supportive care have improved outcomes of severe GAS infections, but continued research efforts are needed to unravel the complexities of these infections and inform evidence-based strategies for prevention, management, and surveillance. Collaboration between clinicians, researchers, and public health authorities is essential to address the evolving landscape of pediatric severe GAS infections and mitigate their effects on child health.

Appendix Additional information about severe group A *Streptococcus* infection among children, France, 2022–2024.
