# Supplementary material for: Severe Group A Streptococcus Infection among Children, France, 2022–2024
Source: Emerg Infect Dis. 2025 Sep;31(9):1698–707. doi: 10.3201/eid3109.250245 (PMC12407216; doi:10.3201/eid3109.250245)
Supplement: Appendix — Additional information about severe group A Streptococcus infection among children, France, 2022–2024. [file 25-0245-Techapp-s1.pdf]

Article DOI: https://doi.org/10.3201/eid3109.250245

EID cannot ensure accessibility for supplementary materials supplied by authors. Readers who have difficulty accessing supplementary content should contact the authors for assistance.

Severe Group A *Streptococcus* Infection among Children, France, 2022–2024

Appendix

Pediatric Data from the French Streptococcus National Reference Center (2012–2019): Invasive *Streptococcus pyogenes* Infections in Children Aged 0 to 18 Years in France

Appendix Table. Clinical features of pediatric invasive GAS infections between 2012 and 2019 (N = 911).

Clinical Features	No. (%)
Age group, y	
<1	133 (14)
1–4	416 (46)
5–9	226 (25)
10–17	135 (15)
Sex	
M	507 (56)
F	404 (44)
Site of infection	
Ear-Nose-Throat	157 (17)
Skin and soft tissue*	214 (23)
Bone and joint	172 (19)
Lower respiratory	137 (15)
Pleuro-pneumonia	64 (7)
Others†	
Streptococcal toxic shock syndrome	117 (13)
Death	78 (8.6)

*Including 41 cases of necrotizing bacterial dermohypodermitis. Varicella was reported in 18% of cases.
†Includes cerebro-meningeal, early neonatal, intra-abdominal, gyneco-obstetrical and endocarditis infections—each representing less than 5% of cases.

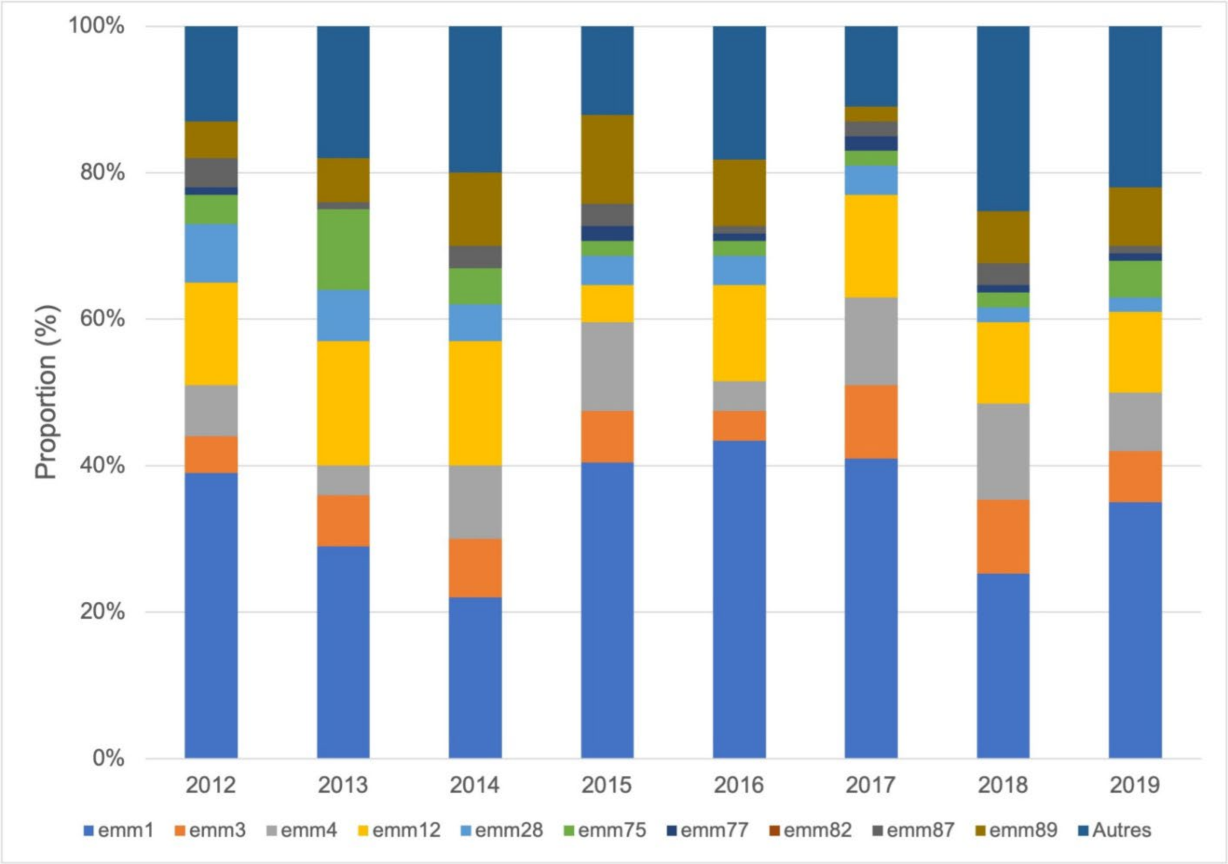

Appendix Figure. Genotype distribution for severe Group A Streptococcus infection among children, by year, France, 2012–2019 (N = 911). The distribution of genotypes reveals a consistent predominance of the emm1 genotype each year. **Note:** This table and graph present data submitted voluntarily to the NRC. The representativeness of these data are estimated at 30% to 40%, depending on the year.
